# Towards a Modular Pathological Tremor Simulation System Based on the Stewart Platform

**DOI:** 10.3390/s23229020

**Published:** 2023-11-07

**Authors:** Jair Fajardo, Leonimer Flávio de Melo

**Affiliations:** 1 Federal Institute of Paraná, Assis Chateaubriand Campus, Assis Chateaubriand 85935-000, Brazil; 2 Department of Electrical Engineering, State University of Londrina, Londrina 86057-970, Brazil; leonimer@uel.br

**Keywords:** tremor simulation, Parkinson’s disease, multibody dynamics, wearable devices, assistive technologies, systems modeling, Kalman filter, sensor fusion

## Abstract

Wearable technologies have aided in reducing pathological tremor symptoms through non-intrusive solutions that aim to identify patterns in involuntary movements and suppress them using actuators positioned at specific joints. However, during the development of these devices, tests were primarily conducted on patients due to the difficulty of faithfully simulating tremors using simulation equipment. Based on studies characterizing tremors in Parkinson’s disease, the development of a robotic manipulator based on the Stewart platform was initiated, with the goal of satisfactorily simulating resting tremor movements in the hands. In this work, a simulator was implemented in a computational environment using the multibody dynamics method. The platform structure was designed in a virtual environment using SOLIDWORKS^®^ v2017 software and later exported to Matlab^®^ R17a software using the Simulink environment and Simscape multibody library. The workspace was evaluated, and the Kalman filter was used to merge acceleration and angular velocity data and convert them into data related to the inclination and rotation of real patients’ wrists, which were subsequently executed in the simulator. The results show a high correlation and low dispersion between real and simulated signals, demonstrating that the simulated mechanism has the capacity to represent Parkinson’s disease resting tremors in all wrist movements. The system could contribute to conducting tremor tests in suppression devices without the need for the presence of the patient and aid in comparing suppression techniques, benefiting the development of new wearable devices.

## 1. Introduction

In the last decades, wearable technologies have been constantly gaining prominence, especially in health-related applications, where miniaturized devices (sensors and actuators) are placed on or near the body, aiming to minimize interference with the user’s activities [1,2]. This is partially due to technological advances in microelectronics, which have allowed the development of miniaturized devices capable of collecting data with high quality in a compact space and with low energy consumption.

As an example, wearable devices designed for tremor suppression can be mentioned. In these projects, sensors and actuators are utilized to actively identify pathological tremors and suppress them, enabling patients to carry out everyday tasks that might have previously been challenging. In the work by Rocon et al. [3], a robotic exoskeleton named WOTAS (wearable orthosis for tremor assessment and suppression) was developed. This system employed DC actuators and adaptive control algorithms to suppress tremors associated with Parkinson’s disease (PD) while allowing users to perform voluntary movements naturally. Another example is the project by Manto et al. [4] called DRIFTS (dynamically responsive intervention for tremor suppression), which aimed to create a wearable prosthesis for actively suppressing PD tremors.

Similarly, in the studies conducted by Herrnstadt et al. [5], wearable prostheses were developed with the objective of suppressing PD tremors. Furthermore, Zhou et al.’s research [6] focused on attenuating tremors caused by PD and proposed devices aimed at reducing this symptom. Generally, these technologies take the form of exoskeletons [3], wristwatch-like devices [7], or even gloves [8,9].

The work by Lora-Millan et al. [10] provides a detailed review of this method, listing 27 projects involving wearable technologies specifically applied to the suppression of pathological tremors. The technologies used in each project, the efficiency of each solution, and the data validation methods employed are indicated, with the latter being the focus of discussion in this study.

During the evaluation of the data in this review article, it was noticed that among the 27 listed works, 20 projects used patients for testing and validation, while 7 projects used validation through electromechanical tremor simulation devices. Regarding the simulators, it was observed that all of them had only 1 degree of freedom of movement. Considering the tremor movements in the wrist, there are six possible movements: adduction, abduction, flexion, extension, supination, and pronation [11], where at least 3 degrees of freedom (3-DOF) would be necessary to perform these movements together.

Considering that using patients in tests involves extremely complex issues, such as ethical committee approvals, adequate space for patient care, availability, and difficulty of volunteers’ participation due to their own comorbidity, resources for sustenance, structural difficulties in small centers, among others, the motivation to use patients in tests for wearable tremor suppression devices comes into question, given the possibility of validation through simulators.

The hypothesis raised is that even in the face of difficulties in conducting tests with humans, the analyses performed naturally are more comprehensive, considering the complexity of real tremor movements. In other words, due to the difficulty in developing equipment that simulates the full complexity of the affected limb’s movement adequately, it is still preferable to conduct tests on humans. The development of an appropriate simulation mechanism could reduce or even eliminate the need for patient testing at various stages. Additionally, there are individual characteristics in each patient’s symptoms, the way tremors present themselves (varying from individual to individual), their progression, or even the use of medications, which are factors that must be taken into account. Thus, even if tests are performed on the same individuals, the temporal space can lead to variable data.

In order to attain a robust variability in the sample space for testing, multiple patients are required, each exhibiting distinct movement characteristics, various stages of the disease, and diverse physical attributes. Consequently, making direct comparisons between techniques to genuinely assess the relative efficacy of each approach becomes a complex task, given the inherent disparities within the datasets.

Addressing this challenge, this study has developed a robotic manipulator model capable of replicating all human wrist movements, thereby facilitating a more authentic simulation of tremor complexity in a realistic manner. In this case study, our objective is to outline the methodology for developing the simulator, assess its workspace, and evaluate its ability to replicate resting tremors associated with PD. With these validated results, it will be possible to subsequently design a robotic mechanism that can enhance the quality of testing during the device development phase and, in certain instances, even obviate the need for prior tremor data collection from afflicted patients. This contribution is anticipated to expedite the advancement of novel wearable technologies aimed at tremor suppression, with reduced direct patient involvement.

While the current focus of this work lies in wrist movements, it holds the potential to extend its capabilities to simulate movements of other body parts, such as the head and feet. As a result, the development of this platform commenced with a simulator designed for the kinematic and dynamic analysis of a 6-DOF parallel manipulator based on the Stewart platform [12,13]. This configuration enables the execution of all distinctive wrist movements. The simulator, even before physical construction, can illustrate its workspace, encompassing both its range and limitations, along with the forces at play in each movement. Additionally, it demonstrates the capability to replicate tremor movements with variations in frequency, amplitude, and positioning within a three-dimensional space. This approach simplifies the determination of optimal requirements for the final physical prototype development.

Given the well-established characteristics of PD tremors, informed by numerous studies, a tangible system can potentially be implemented in the future with the goal of reducing the exposure time of PD patients to tests while still advancing the development of innovative technologies.

### 1.1. Parkinson’s Disease

PD is a progressive neurodegenerative disorder that affects the central nervous system. It is primarily caused by the degeneration of cells that produce the neurotransmitter dopamine, located in the brain region known as the substantia nigra. Dopamine is responsible for transmitting signals between neurons, and its reduction or absence affects certain motor functions. The development of Parkinsonism is preceded by a long prodromal phase, preceding the onset of disease symptoms, with most clinical signs being nonspecific. At the time of initial diagnosis, more than 50% of dopaminergic neurons may have already been lost from the substantia nigra [14].

People with PD often experience symptoms related to apathy, depression, constipation, sleep disorders, problems with posture, balance, and stiffness in movements, as well as loss of smell and cognitive issues. However, the disease is most well-known for its implications on the motor system, resulting in involuntary tremors [15]. It is estimated that the disease affects approximately 1% of people over the age of 60 and up to 4% of people over the age of 80 [16].

### 1.2. Tremor Characterization

The characteristics of pathological tremors are well known in studies and can appear in the arms, legs, and also the head, presenting as rhythmic, involuntary, oscillatory movements with a constant frequency over time and varied amplitude [17]. Most tremors are primarily oscillatory rotations of a body part around a joint or multiple joints. For example, hand tremors can originate from shaky rotation at the wrist, elbow, or shoulder. Similarly, head tremors mainly involve the rotation of the head around the neck [18]. Diagnosis involves a focused approach to patients’ histories and neurological analyses based on the nuances of tremor phenomenology [19]. The main observation is to classify the tremor as an action tremor or a resting tremor [20].

Action tremor occurs during intentional movement of a limb, such as walking or reaching for an object, and can be divided into intention tremor, which manifests when the individual moves toward a target, and a kinetic tremor, which is visible only at the end of the movement toward a target. Postural tremor occurs when a limb remains stretched in a position against gravity, usually occurring at frequencies of 4.5 Hz to 12 Hz [21].

On the other hand, resting tremor occurs involuntarily when the affected body part is completely supported or at rest. The causes can be physical or psychological, but in most cases, it is associated with PD, representing a more severe type of tremor. This symptom typically occurs unilaterally, significantly affecting the hands, and is less frequently diagnosed in the lower limbs [22].

It can be observed that the intensity of resting tremor increases when there is mental effort or when the individual moves another part of the body and can be relieved by voluntary movement of the affected limb. Typically, this tremor is characterized by involuntary movements at frequencies of 3 Hz to 6 Hz, reaching up to 9 Hz in some cases [23]. The amplitude of the tremor is relatively low, ranging from a few millimeters to a few centimeters depending on the severity of the underlying condition, the location of the tremor, and individual factors. It is generally more pronounced in the morning and diminishes as the day progresses. Additionally, the tremor can worsen during moments of stress or anxiety.

Generally, resting tremor is one of the most common and characteristic symptoms of PD, but the amplitude of tremors can vary greatly among patients and may be affected by treatment. Tremor amplitude can be measured using various methods, such as visual observation, accelerometer measurements, and ECGs, among other specific equipment [24]. These methods allow quantifying the tremor amplitude, which is useful for assessing disease progression and treatment efficacy.

Initially, this work focused on representing resting tremor in PD, with frequencies between 3 Hz to 6 Hz, demonstrating the platform’s ability to represent tremor signals at frequencies from 1 Hz to 12 Hz, always considering the maximum stress of the mechanism. The next chapter will detail the methodology of developing the robotic mechanism, and in the Results and Discussion chapter, graphs will be presented with the results of tremor executions on the platform.

## 2. Materials and Methods

The objective of this study is to develop a simulator for a robotic manipulator based on the Stewart platform with the capability to replicate the complexity of PD tremor movements. In this section, we will introduce the simulation system, which allows us to extract data pertaining to the manipulator’s mobility. This includes an examination of its workspace and dynamic behavior, taking into account the interconnected motors, masses, and forces involved. The analysis of these data within the simulation environment will facilitate the subsequent development of a physical platform with greater accuracy and efficiency. In this particular case study, our focus will be on evaluating the requirements associated with resting tremor.

### 2.1. The Stewart Platform

The Stewart platform, as first mentioned by Gough [13] and proposed by Stewart in 1965 [12], is essentially a 6-DOF parallel manipulator, with each actuator responsible for one DOF. There are some structural variants regarding its shape and the configuration of the used actuator [25].

The combination of actuator movements allows for linear and rotational motions. This closed-chain manipulator (parallel actuators) has several advantages when compared to open-chain manipulators (serial actuators), such as high speed, acceleration, load-carrying capacity relative to the motor’s required torque and structural rigidity. These reasons make the Stewart platform excellent for high-precision movements in general [26,27,28].

To conduct a comprehensive study of this platform, it is necessary to develop a model that allows for the evaluation of its kinematics, which depends on the shape, dimensions, and arrangement of structural components. Moreover, understanding its dynamics requires studying the forces impacting the system and their effect on its movement, considering materials, density, mass, center of mass, etc., which can cause positioning errors or undesirable variations in velocity and acceleration [26]. This descriptive and representative model can be structured mathematically. The most popular methods for mathematical implementation are the Newton-Euler methods, which consider the center of mass, components in equilibrium, internal and external forces [29], and the Lagrange method, which evaluates the difference between potential and kinetic energies involved in the model [30]. Both methods can effectively represent the behavior of closed-chain robotic devices, although they have different characteristics in terms of representation and computational complexity.

In this work, the simulation model of the Stewart platform was chosen using the multibody dynamics (MBD) method [31], where each body has its own modeling, and the blocks are connected through joints and ligaments structured based on a developed 3D CAD model. This method intuitively implements the forward kinematic model and the dynamic model, with only the inverse kinematic implementation remaining [32]. The advantage of using this method is the possibility of quickly re-adapting the project when necessary.

#### 2.1.1. Inverse Kinematics

Inverse kinematics is applied to the Stewart platform to determine the optimal positions of the actuators to achieve the desired position. This method is based on mathematical equations that describe the relationship between the positions of the actuators and the final position of the platform. Thus, to perform a given movement, it is necessary to find the linear displacement $l_{i}$ of each prismatic actuator. However, first, the coordinates of the anchor points $B_{i}$ relative to the fixed lower platform and $P_{i}$ relative to the movable platform must be determined.

**Figure 1:**
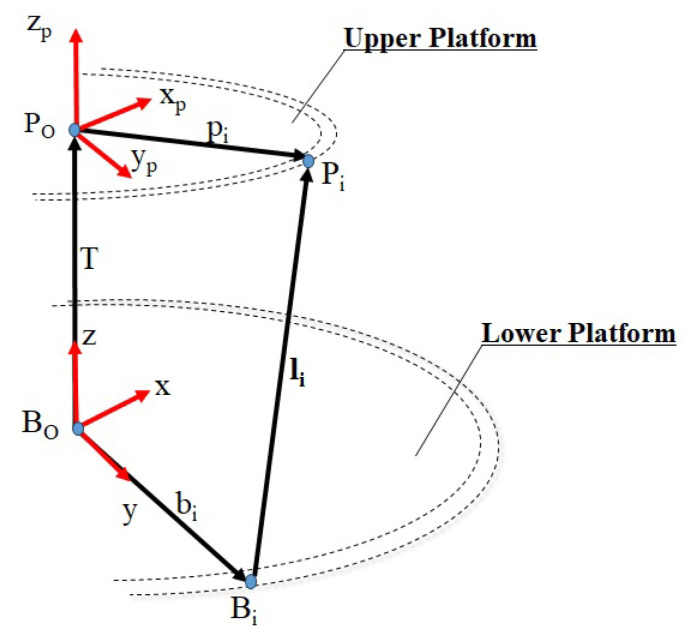
Generalized representation of anchor points and central points of each platform.

In Figure 1, it is possible to observe the origins of each platform, where point $B_{O}={\left[x,y,z\right]}^{T}$ is the central point of the lower platform, with coordinates *x*, *y*, and *z*. Similarly, $P_{O}={\left[x_{p},y_{p},z_{p},\varphi ,\phi ,\psi \right]}^{T}$, where $x_{p}$, $y_{p}$, and $z_{p}$ represent the coordinates of the center of the upper platform, while φ, ϕ, and ψ denote the angles related to wrist movements. Considering that the motion of each actuator influences the position of the end effector of the platform, it is necessary to calculate its orientation $P_{O}$ relative to the base $B_{O}$. Mathematically, this relationship ${}^{P}R_{B}$ is obtained using Euler angles and is achieved through the following sequence of rotations:

(1) $${}^{P}R_{B}=R_{z}\left(\psi \right)\cdot R_{y}\left(\phi \right)\cdot R_{x}\left(\varphi \right)$$

where

$$\begin{array}{c} {}^{p}R_{B}=\left[\begin{array}{ccc} c_{\psi }c_{\phi } & c_{\psi }s_{\phi }s_{\varphi }+c_{\varphi }s_{\psi } & c_{\varphi }s_{\psi }-c_{\varphi }c_{\psi }s_{\phi }\\ -c_{\phi }s_{\psi } & c_{\varphi }c_{\psi }-s_{\varphi }s_{\phi }s_{\psi } & c_{\psi }s_{\varphi }+c_{\varphi }s_{\phi }s_{\psi }\\ s_{\phi } & -s_{\varphi }c_{\phi } & c_{\varphi }c_{\phi } \end{array}\right]\; \end{array}$$

where S=sin and C=cos.

Thus, to determine the extension of the vector $l_{i}$, Equation (2) is used:

(2) $$l_{i}=T{+}^{p}R_{B}\cdot p_{i}-b_{i}$$

**Figure 2:**
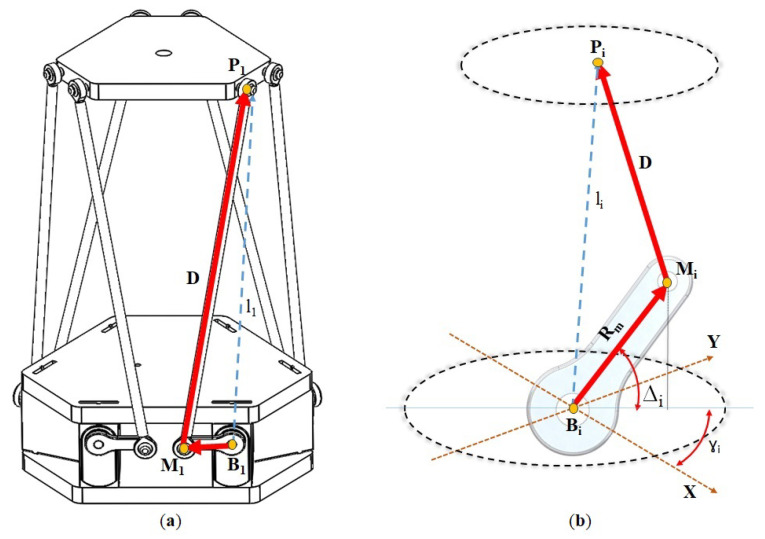
(**a**) Visualization of the position of the links (dotted lines) in the original Stewart platform and the developed model. (**b**) Planar representation of the connecting rods between the lower platform and the upper platform.

The vector *T* represents the translation from the origin $B_{O}$ in the lower platform to the origin $P_{O}$ in the end effector, where $T=\left(t_{x},t_{y},t_{z}\right)\in R^{3}$. One of the junction points of each platform, the vector from $B_{O}$ to $B_{i}$, and the vector from $P_{O}$ to $P_{i}$ are depicted in Figure 1 as vectors $b_{i}$ and $p_{i}$, which are generalized as vectors $b_{1}$ and $p_{1}$ shown in Figure 2a. The vector $l_{i}$ represents the extension of the prismatic actuator, as depicted in Figure 1 and Figure 2.

Finally, to represent the velocities of each actuator, which affect the velocity of the upper platform, the Jacobian matrix is used. The Jacobian matrix is widely used in parallel manipulators and is represented by the following equation:

(3) $$\dot{L}=J\dot{X}$$

where $\dot{L}$ represents the actuator velocity matrix and $\dot{X}$ the velocity of the upper platform.

Both the upper and lower platforms are interconnected by six rotational actuators, each of which is attached to two inflexible rods that transmit the motion from the motors to the upper platform. The aluminum rods are attached to the motor axes with measurements $R_{m}$, which in turn are connected by larger rods composed of two ball joints of the same material and carbon structure, with measurement *D*. The structure used in this project can be seen in Figure 2a. This modification implies the need for the calculation of motion conversion, considering that the Generalized Stewart platform uses a prismatic actuator. Therefore, the prismatic motion will be converted into a rotational motion ${▵}_{i}$ that results in an equivalent motion to the previous $l_{i}$. This composition can be observed in Figure 2b.

To determine the spatial position of the anchor points of the lower platform relative to $B_{O}$, it should be noted that this work utilized rotational actuators instead of the conventionally used prismatic actuators in the generalized platform. Given the use of rotational actuators, it was necessary to supplement the inverse kinematics. Therefore, as depicted in Figure 2b, $R_{m}$ represents the vector from $B_{i}$ = $(x_{i}$  $y_{i}$ $z_{i})$ to $M_{i}$ = $(x_{mi}$ $y_{mi}$ $z_{mi})$, and *D* is the fixed rod length between $M_{i}$ and $P_{i}$ = $(x_{pi}$ $y_{pi}$ $z_{pi})$. Furthermore, $\gamma_{i}$ denotes the angular coordinates of the attachment points, depending on the spatial position where the actuator will be located. Thus, we derive the following equations:

(4) $$R_{m}=R_{mi}=\left|M_{i}-B_{i}\right|$$

(5) $$D=D_{i}=\left|P_{i}-M_{i}\right|$$

The endpoint of vector $M_{i}$ is found through the following transformation:

(6) $$M_{i}={\left(\begin{array}{ccc} x_{mi} & y_{mi} & z_{mi} \end{array}\right)}^{T}{=}^{mi}T_{b}{+}^{mi}R_{b}{\left(\begin{array}{ccc} R_{m} & 0 & 0 \end{array}\right)}^{T}$$

where

(7) $${}^{mi}T_{b}={\left(\begin{array}{ccc} x_{i} & y_{i} & z_{i} \end{array}\right)}^{T}$$

(8) $${}^{mi}R_{b}=R_{z}\left(\gamma_{i}-\frac{\pi }{2}\right)R_{y}\left({▵}_{i}\right)$$

However, the rotation matrix above is only valid for even-indexed servos, as odd-indexed servos have the joints attached on the opposite side. Therefore, to differentiate between the two configurations, it is only necessary to change the sign in the following expression:

(9) $$\left(\begin{array}{c} x_{mi}\\ y_{mi}\\ z_{mi} \end{array}\right)=R_{m}\left(\begin{array}{c} \pm \cos\left({▵}_{i}\right)\sin\left(\gamma_{i}\right)+x_{i}\\ \pm \cos\left({▵}_{i}\right)\cos\left(\gamma_{i}\right)+y_{i}\\ \sin\left({▵}_{i}\right)+z_{i} \end{array}\right)$$

The positive sign in the terms $x_{mi}$ and $y_{mi}$ indicates the valid expression for even-numbered servos, whereas the negative sign is valid for odd-numbered servos. With each new position or orientation of the servos, a new set of vectors $P_{i}$ emerges, and subsequently, the virtual arm $L_{i}$ is obtained, requiring finding the rotation of the motor ${▵}_{i}$ that satisfies it. From the following equations we have:

(10) $$R_{m}^{2}={\left(M_{i}\left({▵}_{i}\right)-B_{i}\right)}^{T}\left(M_{i}\left({▵}_{i}\right)-B_{i}\right)$$

(11) $$D^{2}={\left(P_{i}-M_{i}\left({▵}_{i}\right)\right)}^{T}\left(P_{i}-M_{i}\left({▵}_{i}\right)\right)$$

(12) $$|L_{i}{|}^{2}={\left(P_{i}-B_{i}\right)}^{T}\left(P_{i}-B_{i}\right)$$

For all i∈{1,…,6}, combining the previous equations by Pythagoras leads us to:

(13) $$|L_{i}{|}^{2}-D^{2}+R_{m}^{2}=2{\left(B_{i}-M_{i}\left({▵}_{i}\right)\right)}^{T}\left(B_{i}-P_{i}\right)$$

after substituting with Equation (6), we have that

(14) $$\begin{array}{c} \pm \left(\right|L_{i}{|}^{2}-D^{2}+R_{m}^{2})=2R_{rm}\left(Z_{pi}-z_{i}\right)\sin\left({▵}_{i}\right)\\ +2R_{m}\left[\sin\left(\gamma_{i}\right)\left(x_{pi}-x_{i}\right)-\cos\left(\gamma_{i}\right)\left(y_{pi}-y_{i}\right)\right]\cos\left({▵}_{i}\right) \end{array}$$

Reducing the equation into the following format:

$$\begin{array}{c} L=M\sin{▵}_{i}+N\cos{▵}_{i} \end{array}$$

and using trigonometric identity functions, we have that:

asin(ϕ)+bcos(ϕ)=csin(ϕ+φ)

with,

$$c=\sqrt{a^{2}+b^{2}}$$

and

$$\varphi =\arctan\left(\frac{b}{a}\right)+\left\{\begin{array}{cc} 0, & a\geq 0\\ \pi , & a<0 \end{array}\right\}$$

Taking these considerations into account, we have another sinusoidal function of ${▵}_{i}$ with a phase shift of ς, therefore, we obtain that

$$\begin{array}{c} L=\sqrt{M^{2}+N^{2}}\sin\left({▵}_{i}+\varsigma \right)\\ \sin\left({▵}_{i}+\varsigma \right)=\frac{L}{\sqrt{M^{2}+N^{2}}} \end{array}$$

where

$$\begin{array}{ccc} L & = & |L_{i}{|}^{2}-D^{2}+R_{m}^{2}\\ M & = & 2R_{m}\left(z_{pi}-z_{i}\right)\\ N & = & 2R_{m}\left[\sin\left(\gamma_{i}\right)\left(x_{pi}-xi\right)-\cos\left(\gamma_{i}\right)\left(y_{pi}-y_{i}\right)\right] \end{array}$$

Assuming that *M* is a variable with a positive sign, we can find the angle of the servo ${▵}_{i}$ as follows:

(15) $${▵}_{i}=\arcsin\left(\frac{\pm L}{\sqrt{M^{2}+N^{2})}}\right)-\arctan\left(\frac{N}{M}\right).$$

Thus, it is possible to determine the desired position and orientation of the upper platform if all joints obtain a real solution for all *i*, allowing the complete replacement of all linear motors with rotational motors.

#### 2.1.2. Forward Kinematics and Dynamic Model

Through MBD analysis, it is possible to implement the analysis of forward kinematics and dynamics, combining the design characteristics of each platform component developed in the CAD environment (mass, center of mass, inertia), along with the evaluation of its joints through the interconnection of specific blocks, from which data such as motion, velocity, acceleration, torque, etc., can be extracted.

Considering the nonlinear behavior and complexity of the mechanism, the development process of the simulation system was carried out in two stages. In the first stage, the three-dimensional model was developed in CAD software, as shown in Figure 1. The software used was SOLIDWORKS^®^ v2017, where each platform component was designed separately, respecting the dimensions and materials to be used.

Subsequently, a virtual assembly was performed in the same software, where all parts are attached to each other, and the first evaluation of compatibility between the components was carried out. After this stage, a Simscape multibody link plugin was used to export the fully assembled model from SOLIDWORKS^®^ v2017 into the Simulink environment of Matlab^®^ R17a, generating a preliminary platform model. This integration between the two software reduces simulation time and allows for easy modification of the model. If any physical components need to be altered or adjusted, they can be re-exported through this plugin.

It is also possible to design each element directly within the Matlab^®^ R17a platform. However, the environment does not provide specific CAD tools that assist in the design process. The CAD software environment is more intuitive and allows for greater detailing of parts. In this sense, the SOLIDWORKS^®^ v2017 program maintains a library with specific characteristics of various materials used in this project, which directly influence the dynamic behavior of the platform, as well as its functional characteristics. It is even possible to perform stress tests to improve the dimensioning of the parts.

**Figure 3:**
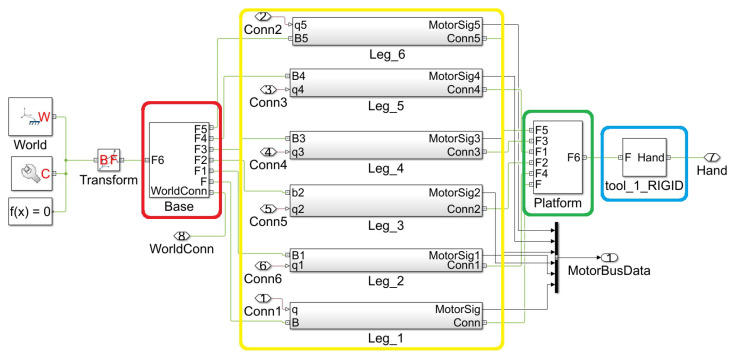
Graphical model used in the modeling of the Stewart platform through MBD. The fixed lower platform block is represented in red, each of the motors and connecting rods in yellow, the movable upper platform in green, and the tool of the model is represented in blue.

The initial model delivered by the plugin in an automatic way is in an *.xml file with a disorganized pre-assembly that requires a review to check for any faults that may have occurred during assembly. However, it accomplishes the task of interconnecting blocks and integrating all joints into the complete model. In Figure 3, it is possible to visualize the simulation model of the platform, now organized and functional, where the four main groups of the platform are enclosed by rectangles.

In the red rectangle, the fixed/inferior platform is represented, which in this case is being used as the reference base body. The yellow rectangle indicates the block composed of connecting rods and joints, and also shows the actuators and sensors. In green, there is the upper platform, followed by the blue rectangle representing the modular tool attached to the platform.

In addition to the interconnection of the blocks, the model is exported with a file extension *.m, which contains data structures that form the characteristics tables of MBD, including material properties such as identification, mass, moment of inertia (MoI), product of inertia (PoI), center of mass (CoM), color, opacity, and others, as well as the data resulting from the transformation operations of each component. This information is directly imported from the model built in the CAD software.

### 2.2. Platform Specifications

The platforms have a hexagonal shape, consisting of both a lower and an upper platform. The complete assembly of the lower platform comprises six independent rotational actuators and two acrylic plates, each with a thickness of 10 mm. These plates are responsible for coupling and securing the motors in place. Consequently, neither of these components actively contributes to the movement of the upper platform. For modeling purposes, the central points of both the lower platform $B_{O}$ and the upper platform $P_{O}$ are situated on cross-sections along the rotational axis of the motors and on the upper platform, respectively. The motor shafts are firmly connected to small aluminum links with a length of Rm. Subsequently, these links are connected via ball joints to rigid carbon rods with a length of *D*, which transmit the motor’s motion to the upper platform.

Regarding the choice of materials for the platforms, acrylic (medium-high impact) with a 10 mm thickness was selected. This material possesses approximately 48% lower specific mass than aluminum while maintaining favorable stiffness and strength properties, as per the specifications provided in the SOLIDWORKS^®^ materials library.

**Figure 4:**
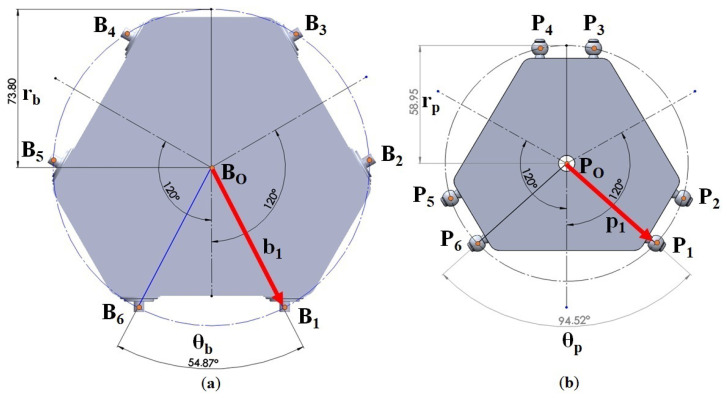
Construction and sizing parameters of the manipulator. (**a**) Lower platform parameters. (**b**) Upper platform parameters.

The specifications and dimensions of the platforms (both upper and lower) used in the study are illustrated in Figure 4. The lower platform has a radius of $r_{b}$ = 73.8 mm and an angle between anchor points $B_{6}$ and $B_{1}$ of $\theta_{B}$ = 54.87°, whereas the upper platform possesses a radius of $r_{P}$ = 58.95 mm and the angle between anchor points P6 and P1 is $\theta_{P}$ = 94.52°. Another crucial dimension pertains to the linkages connecting the actuators to the upper platform, with the linkage attached to the motor having dimensions of Rm = 24 mm and *D* = 214.5 mm. Although these values are fixed for the simulations, they can be adjusted as needed for experimental purposes. A summary of data related to the component masses is provided in Table 1. 

In this work, we sought to replace metallic materials, for the most part, with synthetic materials, due to their lower specific mass contributing to the execution of tremor-inducing movements.

#### 2.2.1. Motor and Control Specifications

The simulation environment allows evaluation in two models. The first one is based on an ideal motor, where it is possible to extract information regarding static and dynamic forces involved in a particular position or movement, considering the direction and sense in which gravity acts on the platform, material mass, inertia, platform structure, and the tool attached to the upper platform. This study is essential for defining the specifications of the actuators required for the operation of a potential real platform to be developed, enabling the evaluation of motor requirements for a specific action or function.

**Figure 5:**
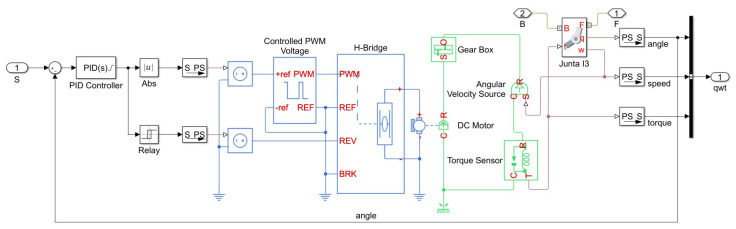
Model of the DC motor used and integrated in the Simulink environment.

The second simulation approach involves using a pre-specified actuator, enabling the study of motion and forces given a particular motor. This simulator allows for the simulation of BLDC motors, stepper motors, and DC motors. For this case study, DC motors were considered, as shown in Figure 5. Generic parameters for a DC motor were used for the simulation, and the model was applied to each of the six motors on the platform. With the motor modeling, a control system is required. In this case, a PID (proportional–integral–derivative) controller was used, responsible for correcting the actuator’s position based on the required setpoint position in comparison to the current position of the motor ${▵}_{i}$.

#### 2.2.2. Modular Tool Coupling

**Figure 6:**
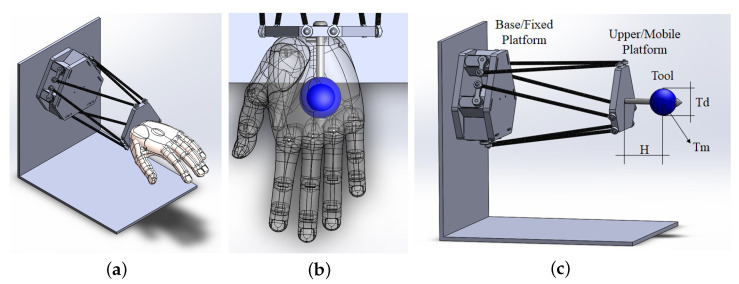
View of the Stewart platform configuration from different perspectives. (**a**) A human hand-shaped tool attached. (**b**) The hand-shaped tool being replaced by a sphere-shaped tool. (**c**) Representation of the dimensions of the modular tool.

Considering the potential for utilizing this platform in a variety of tests, there is the option to couple, exchange, and reconfigure modular tools on the upper platform. Therefore, a modular tool was added to simulate the mass of the human hand in this study, as illustrated in Figure 6. The modular tool installed on the upper platform comprises an adjustable-length linkage, essentially a configurable mass sphere attached to an adjustable distance rod. It is important to note that different tools can be coupled based on project requirements, allowing for the addition of complementary masses, specific sensors, or even flexible components. The specifications and shape of the tool will be determined exclusively by the specific application in each project. In this work, the sphere was used as the mass reference, with the mass of the attached rod being disregarded.

According to Im, Ellen E. et al.’s article [33], it is observed that, although the weight of a limb may vary due to factors such as ethnicity and age, the human hand generally accounts for an average of 0.7% of an individual’s body weight. In other words, for an individual weighing 80 kg, the hand represents a mass of approximately 0.448 kg. Therefore, for this study, the linkage will have a length of *H* = 100 mm, and its mass has been neglected. The sphere depicted in Figure 6, representing the mass of the human hand, has a diameter of Td = 40 mm and a mass of Tm = 450 g. While these values are fixed for the simulations, they can be adjusted as needed for specific experiments.

Another important aspect to consider is the ability to modify the direction and orientation of gravity concerning the platform, enabling the evaluation of the robotic limb in different positions relative to the ground. In this project, which aims to simulate a resting wrist scenario, a horizontal position was selected to represent a seated patient with their wrists supported on an armrest, with gravity acting perpendicular to the platform.

**Figure 7:**
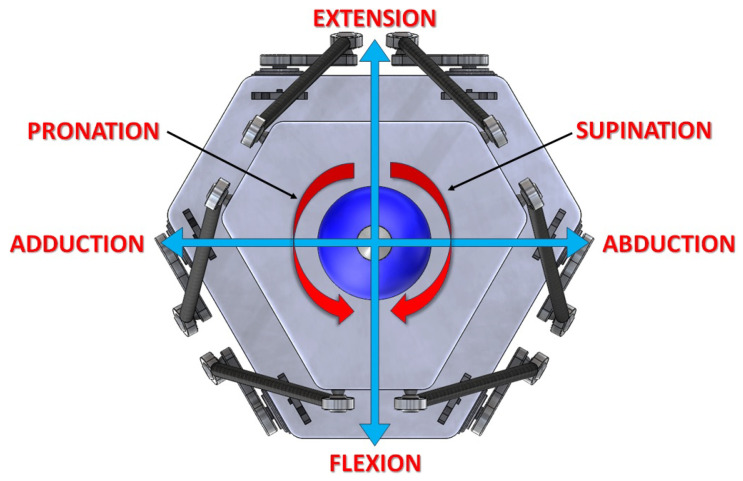
Demonstration in a front view of the wrist movements that will be performed by the platform in the simulator.

For a better understanding and comparison between wrist movements and platform movements, from this point in the text, the yaw (φ), pitch (ϕ), and roll (ψ) motions will directly correspond to abduction/adduction, extension/flexion, and supination/pronation movements of the wrist, respectively, as illustrated in Figure 7.

### 2.3. Workspace Evaluation

The study of the Stewart platform’s workspace can be divided into three methodological classes. The first method is the Jacobian method [34], in which workspace limits are defined as singular Jacobian sets. The second method [27] differs from the first, as the workspace is determined by an algorithm that allows a graphical representation of the reachable regions in two or three dimensions in Cartesian space. Finally, the discretization method is the model used as the basis for this work. Essentially, this model determines the definition of a specific workspace region, and then each point in this region is checked by the platform incrementally to verify which points actually belong to the robot’s workspace.

Structurally, the dimensions of the upper and lower platforms, joint constraints, dimensions of the connecting rods, and tool dimensions have a direct impact on the manipulator’s mobility and are crucial for assessing the robot’s workspace. This evaluation is of utmost importance because the simulation establishes mechanical boundaries and detects potential collisions between components during motion. It becomes essential to verify if the designed mechanism can faithfully replicate PD tremors, closely resembling reality. It is fundamental to ascertain whether the range of motion performed by the model aligns with the necessary amplitude to represent tremor movements, both in terms of inclination and spatial displacement.

According to Markulov [11], the wrist has some limitations within its range of motion. Following the anatomy of the wrist, the movements of extension and flexion are the most affected by resting tremors.

To enable the platform to traverse the entire workspace, a function with a spherical spiral design was used, based on the following Equations (16)–(18) below:

(16) $$\begin{array}{ccc} \varphi & = & \left(a+bt)sin(t\right) \end{array}$$

(17) $$\begin{array}{ccc} \phi & = & \left(a+bt)cos(t\right) \end{array}$$

(18) $$\begin{array}{ccc} \psi & = & A\sin(2\pi f) \end{array}$$

**Figure 8:**
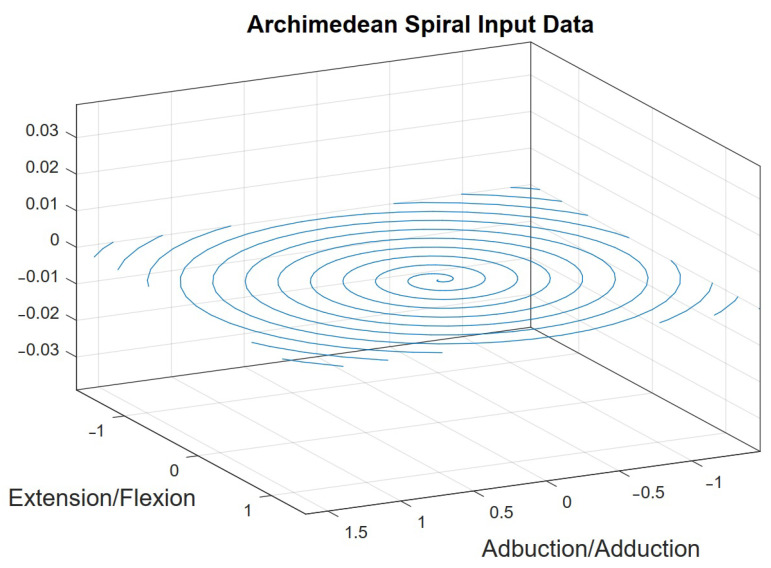
Two-dimensional input signal in the form of an infinite spiral.

Known as the Archimedean spiral, the input represented in Figure 8 was used as the basis for the study of the workspace and applied in performing spherical movements, considering that the tremor movements in the wrist only involve spherical and rotational motions.

The variable *a* represents the center of the spiral, *b* determines the distance factor between each rotational interaction, and *t* determines the rotation angle. As *t* increments, the spiral shape is formed. The values of $x_{p}$, $y_{p}$, and $z_{p}$ in this evaluation were considered constant:

(19) $$P_{O}={\left[\begin{array}{cccccc} x_{p} & y_{p} & z_{p} & \varphi & \phi & \psi \end{array}\right]}^{T}$$

For each new unique point generated by Equations (16) and (17), the platform’s rotation limits with predetermined values (max≥A≥min) were verified by executing a sinusoidal signal as shown in Equation (18), where the variable *A* determines the amplitude of the rotational movement in one of the directions (clockwise or counterclockwise). This allows for the analysis of the 3-DOF in a two-dimensional graph enhanced by a color map. The graph illustrating the progression of movements based on input Equations (16)–(18) can be found in the results section.The platform’s orientation is in the horizontal position, with the force of gravity acting in the direction of wrist flexion, as shown in Figure 6.

### 2.4. Processing Data of Real Parkinson’s Tremor Movements

In this test, a real PD tremor signal will be used, encompassing tremor frequencies, resonance or harmonic frequencies, and all three axes of movement acting together. It is essential to note the presence of noise in the captured signals, as well as interference from the sensors themselves in the patients’ tremor movements. As a result, this simulation becomes more realistic in replicating PD tremors.

The data were collected from a study [35] and kindly provided for this work. Originally, 18 PD patients participated in this study (18 individuals, 8 women, and 10 men), aged between 48 to 73 years. For data acquisition, the MetaMotionC sensor was used, equipped with a three-axis gyroscope and accelerometer (16 bits, ±2000◦/s, ±16 g). The patients were seated in a relaxed position to highlight the data related to resting tremor.

**Figure 9:**
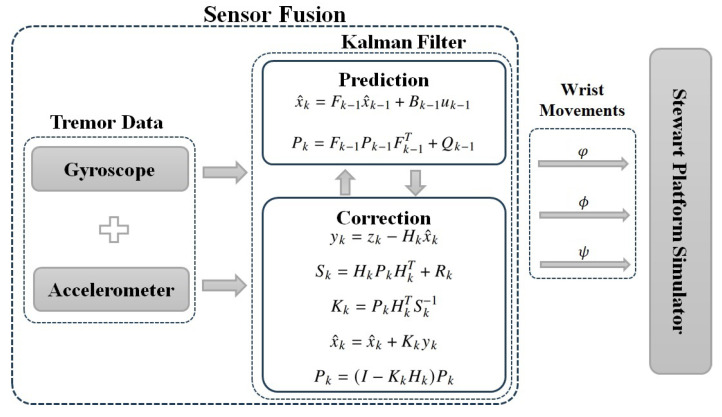
Sequence of sensor data fusion and processing using the Kalman filter.

According to Wang et al. [36], the principle of measuring the rotation angle of a movable surface is equivalent to measuring the rotation angle of a rigid body rotating around a fixed axis. In this way, from the sensor information, the Kalman filter (KF) was applied to fuse the sensor data and convert the acceleration and angular velocity signals into wrist inclination signals, making it possible to execute them as inputs to the simulator as shown in Figure 9.

Proposed by Rudolf Kalman in 1960 [37], the KF is an algorithm used to obtain accurate estimates of a system’s state, even when that state is partially observed and subject to uncertainties. Among the characteristics of the KF, one can mention its ability to optimally combine past and present information, taking into account the uncertainties associated with a plant even without a precise mathematical model. With wide applications in engineering, navigation, robotics, and signal processing, the KF employs a mathematical model that describes the system’s dynamics and performs data fusion from different sensors, resulting in more reliable data as the final output. Therefore, it is particularly effective when measurements are subject to noise and when more accurate estimates are desired with a limited number of observations.

The filter algorithm consists of two main steps: the prediction step and the correction step. Figure 9 also illustrates the two-step algorithm, where two time instants are used and indicated by the index: time instant *k*, representing current information, and time instant k−1, representing retained information from a past state. In the prediction step, the mathematical model of the system and an estimation of the previous state are used to predict the current state of the system. This prediction is accompanied by an estimation of the uncertainty associated with it. Matrices *P* and *Q* represent both the estimated covariance and process noise. The variable *u* represents the input vector, $\hat{x}$ represents the estimated state, $F_{k-1}$ denotes the state transition matrix from time instant k−1 to *k*, and *B* signifies the system input matrix.

In the update step, the filter incorporates recent observations to refine the estimation of the current state of the system. It combines the previous prediction with the observed information, taking into account the uncertainties in both the prediction and the measurements. Through statistical calculations, the KF adjusts the weighting of this information to obtain a more accurate estimate and reduce uncertainty, now with the index ${}_{k}$ referring to the current state. The observed measurement is represented by *z*, *H* is the state-to-measurement observation transition matrix, *R* is the covariance of the measurement noise, *y* is the innovation, and *S* is its covariance. The Kalman gain *K* is calculated to minimize the state estimation error of the system. It takes into account $P_{k}$ and $R_{k}$, thus determining how much weight should be given to the measurements compared to the prediction in the state estimate update.

Regarding the models used, Equation (20) was considered for the accelerometer, and Equation (21) was used for the gyroscope, as stated below:

(20) $$\alpha_{acc}=\alpha_{external}-g+b_{a}+n_{a}$$

(21) $$\omega_{gyro}=\omega +b_{g}+n_{g}$$

Additionally, in this algorithm, the following values were utilized: dt = 0.01, Qa = 0.001, $Q_{bias}$ = 0.003, and *R* = 0.03, as follows:

(22) $$F=\left[\begin{array}{cc} 1 & -{▵}_{t}\\ 0 & 1 \end{array}\right]$$

(23) $$B=\left[\begin{array}{c} {▵}_{t}\\ 0 \end{array}\right]$$

(24) $$Q=\left[\begin{array}{cc} Q_{a} & 0\\ 0 & Q_{bias} \end{array}\right]$$

The KF is computationally efficient, making it suitable for real-time implementations. As a result of the KF’s output, the angles related to wrist movements, including the rotation angle, are obtained. Thus, the data are ready to be used as input for the system represented by Equation (19).

### 2.5. Evaluative Metrics

After executing the input data through the simulator, several metrics were used to evaluate the platform’s behavior during the execution of real data. Graphs were overlaid in the time and frequency domains, a scatter plot was presented to assess the correlation between the data, and, finally, to identify the platform’s ability to reproduce the input signals, the Pearson Correlation Coefficient was used.

The Pearson correlation is a statistical metric that evaluates the degree of linear relationship between two sets of data, resulting in a number that can range from −1 to 1. A correlation coefficient of 1 represents a signal that is fully correlated with the original signal, 0 represents no correlation, and −1 indicates an inverse correlation. Therefore, it is expected that the output signals from the simulator will be close to 1, indicating that the platform has good simulation capability for the input tremor signals. Basically, the calculation of the Pearson Correlation Coefficient is based on Equation (25), allowing the calculation of the coefficient between the two data sets:

(25) $$r=\frac{\sum_{i=1}^{n}\left(x_{i}-\bar{x}\right)\left(y_{i}-\bar{y}\right)}{n\cdot \sigma_{x}\cdot \sigma_{y}}$$

where *r* is the Pearson correlation coefficient. Regarding the evaluated dataset, $x_{i}$ and $y_{i}$ are the corresponding reference values, $\bar{x}$ and $\bar{y}$ refer to the means, *n* is the number of data elements, and finally, $\sigma_{x}$ and $\sigma_{y}$ are the standard deviations.

## 3. Results and Discussion

In this analysis of the results, the data extracted from the simulator executions were evaluated, emphasizing the possibility of studying the requirements imposed by the project so that the platform meets the necessary specifications for its operation. It is worth noting that, at this stage, the definitive specifications are not intended to be obtained, but rather to observe the possibilities and advantages that the simulator can bring in aiding the choice of materials and dimensions for the real platform to be developed. Thus, the development of the simulator aims to reduce costs and implementation time for a real platform in the future.

### Workspace Execution

**Figure 10:**
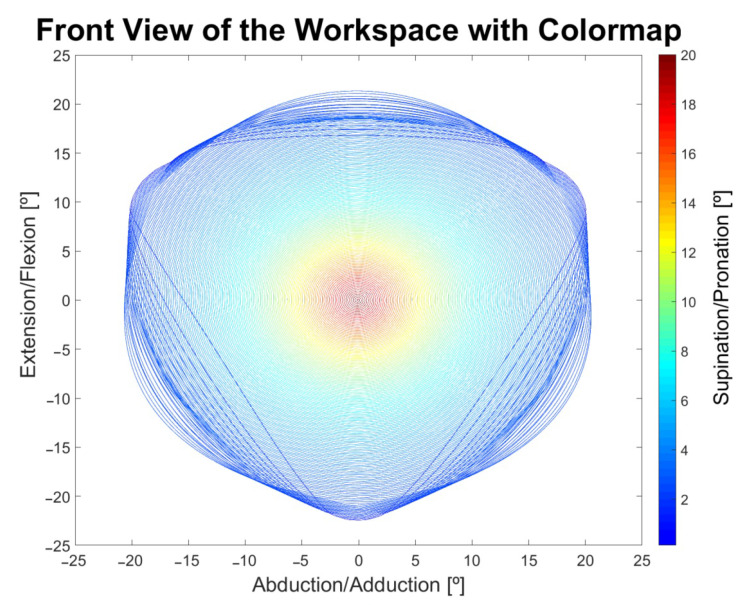
Frontal view of the workspace. Darker colors represent the manipulator’s capability to perform SU/PR movement.

With the aim of visualizing the mechanical limits of the platform, the simulator was used with an ideal motor, as the forces involved in the movement were not evaluated at this stage. In the graph of Figure 10, the complete workspace in two dimensions can be observed, considering the perspective of the frontal plane. The color scheme of the graph defines the maximum range of platform rotational mobility, where darker colors represent a greater capacity for supination/pronation, while lighter colors indicate lower rotational mobility at a specific position. It can also be observed that in the space where there is a high degree of inclination, both for extension/flexion and abduction/adduction, there is a lower capacity of the platform to perform the wrist rotation supination/pronation movement. This is due to the fact that the high degree of inclination of the platform puts more stress on the limits of some actuators, preventing the movement of other DOFs. However, this is an expected design characteristic of the Stewart platform itself.

Considering the limits of the joints, the closer the platform is to the center, the greater its capacity for supination/pronation rotation, with a limit of ±21.1°. The increase in the diameter of the spiral in the input function results in more oblique inclinations, considering that the two-dimensional input from Figure 10 was inserted into a spherical coordinate system, limiting it to approximately ±20.8° for both supination/pronation and abduction/adduction, totaling an inclination capacity of ±21.6° for both DOFs.

**Figure 11:**
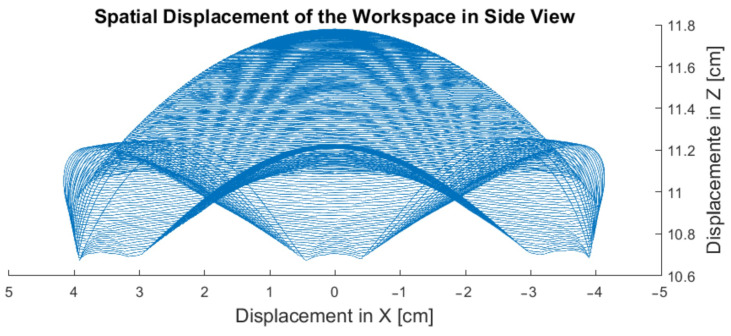
Side view display of workspace displacement.

In Figure 11, it is also possible to visualize the workspace from a spatial perspective in terms of displacement. It demonstrates the spatial displacement of the modular tool attached to the upper platform from a side view of the three-dimensional workspace. The displayed displacement is proportional to the length of the tool used. It can be observed that when attempting to exceed the limits of abduction/adduction and extension/flexion movements, the upper platform starts to exhibit movement errors. This behavior is due to the mechanical limitations that are inherent in the developed design.

The work by Matsumoto et al. [38] indicates that patients with severe PD exhibit hand tremor movements with an inclination amplitude of approximately 10 cm during flexion/extension movements. This suggests that despite the wrist having a wide range of motion, rest tremors do not fully utilize the entire workspace. Therefore, simulations of PD tremors can use a smaller workspace.

Figure 11 demonstrates the spatial displacement that the tool undergoes as it moves within its workspace. It appears that the tool’s displacement is smaller than what was previously suggested for severe tremors. However, it is important to note that the total displacement referred to in Matsumoto et al.’s article [38] is measured from the fingertips. In the case depicted in Figure 11, this measurement is taken from the center of mass of the hand, which has a displacement of 10 cm from the upper platform. If, for instance, a hand with a dimension of 15 cm is considered, this displacement could easily exceed that of severe tremors. Furthermore, even if the simulator does not achieve the necessary value to accurately represent the spatial displacement of severe tremors, it would still serve its purpose of suggesting a review of the platform’s design parameters.

#### Simulating Parkinson’s Disease Tremors

Starting the tests with the generic motor, the simulator now incorporates the model shown in Figure 5, where experiments will be conducted to evaluate the platform’s behavior. Also present, in addition to the motor model, is the PID controller used in the simulation within the Simulink environment. The parameters used in the motor specification are represented in Table 2. For tuning the PID parameters, the Ziegler–Nichols method was used [39].

**Figure 12:**
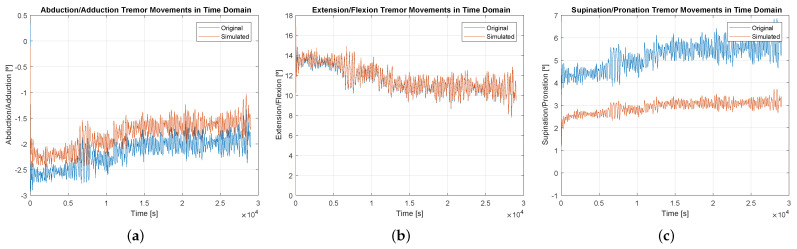
Time domain graphs related to the wrist tilt angle of the right hand of patient 2. (**a**) Abduction/adduction movements. (**b**) Extension/flexion movements. (**c**) Supination/pronation movements.

The data obtained from the study, converted into wrist inclination data using the KF, were used as input for the platform, resulting in the information displayed in Figure 12. This graph spatially represents a sample of the tremor input. A total of 20 samples were executed from 10 patients, with data collected from both the right and left hands. In this testing phase, the platform’s ability to perform PD tremor movements in all three axes of motion was evaluated.

Figure 12 depicts graphs illustrating the executed tremor movements superimposed on the motion graph of the simulator. Visually, it is evident that there are similarities between the signals in all axes of movement. However, there is a deviation between the input and output data in the time domain, particularly in the supination and pronation movement (Figure 12c). This deviation can arise from various factors, including suboptimal PID controller parameters, excessive joint play, the chosen motor potentially being unsuited for the task, design issues (such as excess weight of components), operating in an unsuitable region of the workspace, and other possibilities. Nonetheless, it is still feasible to execute tremor movements adequately, as evidenced in the subsequent results.

**Figure 13:**
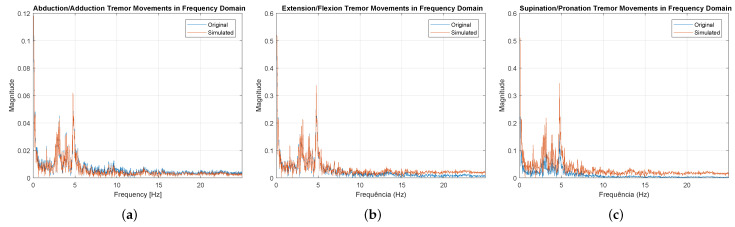
Frequency domain graphs related to the right hand of patient 2. (**a**) Abduction/adduction movements. (**b**) Extension/flexion movements. (**c**) Supination/pronation movements.

In Figure 13, the similarity between the signals is also displayed in the frequency domain. In this domain, it can be observed that the characteristics of PD were preserved, where the dominant frequencies are generally in the range of 3 Hz to 6 Hz, and can reach up to 9 Hz.

The third metric to be observed is the scatter plot. In this graph, the points vary from a dark shade (blue) to a lighter shade (yellow) representing the passage of time during the movements. Darker points represent initial movement data, showing greater dispersion. This occurs because the platform starts at a different point from the starting point of the patient, and this initial point is random. On the other hand, the lighter scatter points represent data at a more advanced time, demonstrating a high degree of linearity between the samples. In other words, the data are highly correlated.

**Figure 14:**
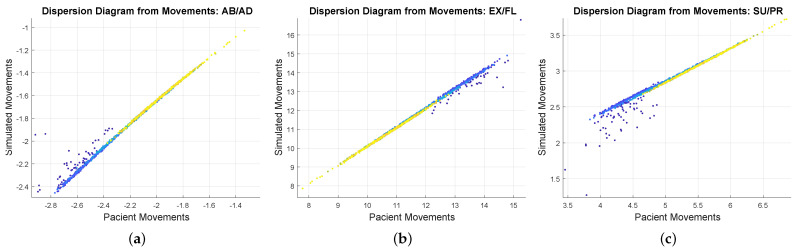
Scatter plot relative to the movement of the patient and the simulator, where the variation in shade of colors represents time, starting from the blue tone. (**a**) Abduction/adduction (AB/AD) movements. (**b**) Extension/flexion (EX/FL) movements. (**c**) Supination/pronation (SU/PR) movements.

Evaluating the Pearson correlation coefficient, the values obtained were 96.53% for supination/pronation, 99.46% for extension/flexion), and 96.66% for abduction/adduction, demonstrating a strong correlation between the real and simulated signals. It should also be noted that the data presented in Figure 12, Figure 13 and Figure 14, refer to only the right hand of one of the 10 participants.

**Figure 15:**
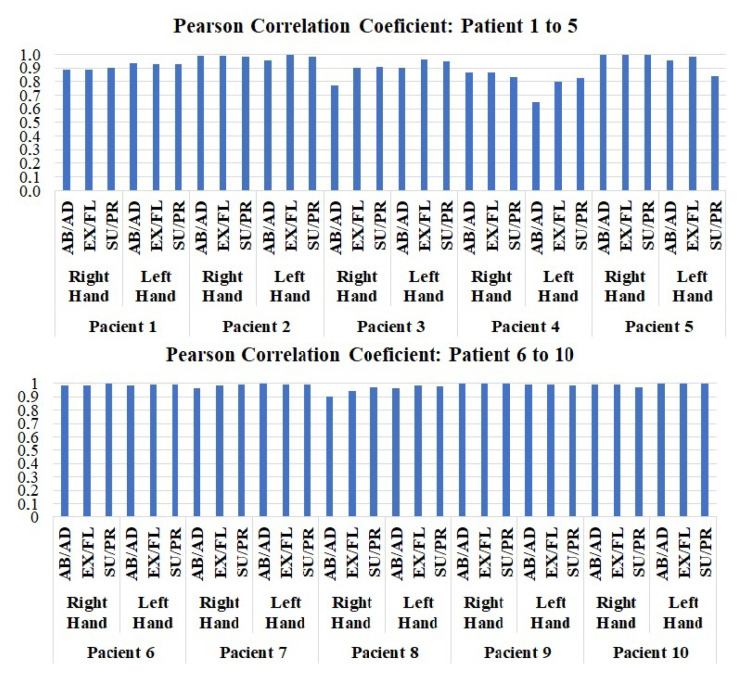
Data of the Pearson correlation coefficient for all data samples from patients with tremor symptoms. Abduction/adduction (AB/AD), extension/flexion (EX/FL), and supination/pronation (SU/PR) movements.

It should be emphasized that the same tests were reproduced for all patients. In the graph represented in Figure 15, the Pearson correlation coefficient was calculated for all patient samples, and the overall average remained above 0.9474. In some cases, the coefficient fell below 0.8, such as in the abduction/adduction movement of the left hand of patient 4, and also in the same movement of the right hand of patient 3. In these specific cases, patients exhibited relatively low amplitudes in these movements when compared to their own hands or even those of other patients. This characteristic may occur in patients who do not exhibit bilateral characteristics in their tremor symptoms, or alternatively, the tremor in these patients shows lower intensity in certain movements. Thus, the platform was unable to accurately represent the signals of these components.

Nevertheless, Pearson correlation coefficient values above 0.9 represent a high degree of correlation. Thus, the platform successfully replicated PD tremors, even considering the noise and uncertainties during data capture and conversion, the tremor characteristics remained. Therefore, the simulated signal truly represents the resting tremor of a patient with PD, even considering the current settings of the platform listed earlier.

The simulation system serves as an excellent tool for constructive testing, such as evaluating the workspace, determining optimal parameters for motors, and conducting dynamic tests and analyses. Furthermore, it enables operational tests, performing specific movements, and testing the platform’s performance. Defining the minimum requirements for choosing a motor or specifying the dimensional parameters of the mechanical components of the platform are the main contributions that the simulation system can provide. For the case study of this work, the application of simulating PD tremors allows for an expansion in testing wearable device prototypes, avoiding the maximum use of debilitated patients but without losing the characteristics of pathological tremors during tests.

## 4. Conclusions

This work aimed to present a simulation system of a robotic manipulator based on the Stewart platform. The platform aims to simulate tremors in the wrist with characteristics similar to those found in PD, allowing for more in-depth testing and studies of wearable technologies capable of reducing this problem in patients. With the simulator, it was possible to evaluate the studied model based on its workspace, aiming to identify the mechanical limits of the platform, as well as the simulation of real tremor movements of PD. In this work, generic constructive data of a direct current motor were used, considering that the main objective was not to test the object itself, but rather to present some of the possibilities of studies. The simulator fulfilled its function, demonstrating that it is indeed capable of replicating PD tremors with high precision and good accuracy, also allowing for improvements that can be made in the robotic system, avoiding execution costs and analyzing errors. This preliminary evaluation will help reduce costs in purchasing materials and provide predictability of the behavior of the real mechanism to be implemented.

There is also the possibility of manipulating patient data through signal processing, applying filters, adding noise, and modifying signal amplitudes to simulate a reduction or increase in symptom severity. Modifying frequency spectra, evaluating harmonics, and generating purely synthetic signals. Economic advantages can also be included, reducing the development time of technologies and addressing ethical concerns by exposing the patient to fewer tests during the research and development phase of a product.

With the implemented simulation tool, the next step will be to develop an actual platform, expanding the range of testing possibilities and the applicability of the mechanism. This includes broadening the types of pathological tremors covered by the simulator and even exploring the simulation of other human body parts.

## Figures and Tables

**Table 1:** Components and mass parameters.

Stewart Platform Simulator	
**Components**	**Mass**
Motor Link (Rm)	6.26 g
Platform Link (*D*)	37.14 g
Upper Platform	95.24 g
Tool Link	0 g (not considered)
Tool Mass (Tm)	450 g

**Table 2:** Motor and control Simulink parameters.

Motor Parameters	
**Parameter**	**Value**
Armature Resistance (Ra)	1.0012 Ω
Armature Inductance (La)	1.201 × $10^{-6}$ H
Torque Constant (Kt)	0.0208 N · m/A
Rotor Inertia (*J*)	0.42 × $10^{-9}$ Kg · m^2^
Rotor Damping (Iam)	0.82 × $10^{-11}$ N·m/(rad/s)
Gear Ratio	10
PID Controller	$K_{p}$ = 20, $K_{i}$ = 4, $K_{d}$ = 8

## Data Availability

The data underlying the results presented in this paper are not publicly available at this time, but may be obtained from the authors upon reasonable request.

## References

[1] Mao P., Li H., Yu Z. (2023). A Review of Skin-Wearable Sensors for Non-Invasive Health Monitoring Applications. Sensors.

[2] Farhani G., Zhou Y., Jenkins M.E., Naish M.D., Trejos A.L. (2022). Using Deep Learning for Task and Tremor Type Classification in People with Parkinson’s Disease. Sensors.

[3] Rocon E., Ruiz A., Brunetti F., Pons J., Belda-Lois J., Sanchez-Lacuesta J. On the use of an active wearable exoskeleton for tremor suppression via biomechanical loading. Proceedings of the 2006 IEEE International Conference on Robotics and Automation, 2006. ICRA 2006.

[4] Manto M., Topping M., Soede M., Sanchez-Lacuesta J., Harwin W., Pons J., Williams J., Skaarup S., Normie L. (2003). Dynamically responsive intervention for tremor suppression. IEEE Eng. Med. Biol. Mag..

[5] Herrnstadt G., McKeown M.J., Menon C. (2019). Controlling a motorized orthosis to follow elbow volitional movement: Tests with individuals with pathological tremor. J. NeuroEng. Rehabil..

[6] Zhou Y., Naish M.D., Jenkins M.E., Trejos A.L. (2017). Design and validation of a novel mechatronic transmission system for a wearable tremor suppression device. Robot. Auton. Syst..

[7] Tabacof L., Braren S., Patterson T., Fry A., Putrino D. (2021). Safety and Tolerability of a Wearable, Vibrotactile Stimulation Device for Parkinson’s Disease. Front. Hum. Neurosci..

[8] Zhou Y., Jenkins M.E., Naish M.D., Trejos A.L. Impact of suppressed tremor: Is suppression of proximal joints sufficient?. Proceedings of the 2018 IEEE EMBS International Conference on Biomedical & Health Informatics (BHI).

[9] Awantha W., Wanasinghe A., Kavindya A., Kulasekera A., Chathuranga D. A Novel Soft Glove for Hand Tremor Suppression: Evaluation of Layer Jamming Actuator Placement. Proceedings of the 2020 3rd IEEE International Conference on Soft Robotics (RoboSoft).

[10] Lora-Millan J.S., Delgado-Oleas G., Benito-León J., Rocon E. (2021). A Review on Wearable Technologies for Tremor Suppression. Front. Neurol..

[11] Omarkulov N., Telegenov K., Zeinullin M., Tursynbek I., Shintemirov A. Preliminary mechanical design of NU-Wrist: A 3-DOF self-aligning Wrist rehabilitation robot. Proceedings of the 2016 6th IEEE International Conference on Biomedical Robotics and Biomechatronics (BioRob).

[12] Stewart D. (1965). A Platform with Six Degrees of Freedom. Proc. Inst. Mech. Eng..

[13] Gough V. (1957). Contribution to discussion to papers on research in automobile stability and control and in tire performance. Proc. Automot. Div. Inst. Mech. Eng..

[14] Lee J.Y., Martin-Bastida A., Murueta-Goyena A., Gabilondo I., Cuenca N., Piccini P., Jeon B. (2022). Multimodal brain and retinal imaging of dopaminergic degeneration in Parkinson disease. Nat. Rev. Neurol..

[15] Lana R.C., Álvares L., Nasciutti-Prudente C., Goulart F.R.P., Teixeira-Salmela L.F., Cardoso F.E. (2007). Percepção da qualidade de vida de indivíduos com doença de parkinson através do PDQ-39. Rev. Bras. Fisioter..

[16] de Lau L.M.L., Breteler M.M.B. (2006). Epidemiology of Parkinsons disease. Lancet Neurol..

[17] Anouti A., Koller W.C. (1995). Tremor disorders. Diagnosis and management. West. J. Med..

[18] Elble R.J., McNames J. (2016). Using Portable Transducers to Measure Tremor Severity. Tremor Other Hyperkinetic Mov..

[19] Louis E.D. (2016). Diagnosis and Management of Tremor. Contin. Lifelong Learn. Neurol..

[20] Louis E.D. (2019). Tremor. Contin. Lifelong Learn. Neurol..

[21] Sabra A.F., Hallett M. (1984). Action tremor with alternating activity in antagonist muscles. Neurology.

[22] Abboud H., Ahmed A., Fernandez H.H. (2011). Essential tremor: Choosing the right management plan for your patient. Clevel. Clin. J. Med..

[23] Deuschl G., Papengut F., Hellriegel H. (2012). The phenomenology of Parkinsonian tremor. Park. Relat. Disord..

[24] Haubenberger D., Abbruzzese G., Bain P.G., Bajaj N., Benito-León J., Bhatia K.P., Deuschl G., Forjaz M.J., Hallett M., Louis E.D. (2016). Transducer-based evaluation of tremor. Mov. Disord..

[25] Szufnarowski F. (2013). Stewart Platform with Fixed Rotary Actuators: A Low Cost Design Study. Adv. Med. Robot..

[26] Furqan M., Suhaib M., Ahmad N. (2017). Studies on Stewart platform manipulator: A review. J. Mech. Sci. Technol..

[27] Kang B., Chu J., Mills J.K. (2001). Design of high speed planar parallel manipulator and multiple simultaneous specification control. Proceedings of the 2001 ICRA, IEEE International Conference on Robotics and Automation (Cat. No. 01CH37164).

[28] Tsai L.W. (1999). Robot Analysis: The Mechanics of Serial and Parallel Manipulators.

[29] Dasgupta B., Mruthyunjaya T.S. (1998). Closed-Form Dynamic Equations of the General Stewart Platform through the NewtonEuler Approach. Mech. Mach. Theory.

[30] Bingul Z., Karah O. (2012). Dynamic Modeling and Simulation of Stewart Platform.

[31] Kane T.R., Levinson D.A. (1983). Multibody dynamics. ASME J. Appl. Mech..

[32] Shabana A.A. (2020). Dynamics of Multibody Systems.

[33] Im E.E., Stewart I.J., Morrow B.D., Tilley M.A., Heegard K.D., Aden J.K., Chung K.K., Cotant C.L. (2012). Retrospective Review of Serum Creatinine and Creatinine-Based Measures of Estimated Glomerular Filtration Rate in an Amputee Population. Mil. Med..

[34] Spencer S.J., Klein J., Minakata K., Le V., Bobrow J.E., Reinkensmeyer D.J. A low cost parallel robot and trajectory optimization method for wrist and forearm rehabilitation using the Wii. Proceedings of the 2008 2nd IEEE RAS & EMBS International Conference on Biomedical Robotics and Biomechatronics.

[35] de Araújo A.C.A., da Rocha Santos E.G., de Sá K.S.G., Furtado V.K.T., Santos F.A., de Lima R.C., Krejcová L.V., Santos-Lobato B.L., Pinto G.H.L., dos Santos Cabral A. (2020). Hand Resting Tremor Assessment of Healthy and Patients With Parkinson’s Disease: An Exploratory Machine Learning Study. Front. Bioeng. Biotechnol..

[36] Wang C., Tu X., Chen Q., Yang Q., Fang T. (2022). Movable Surface Rotation Angle Measurement System Using IMU. Sensors.

[37] Kalman R.E. (1960). A New Approach to Linear Filtering and Prediction Problems. J. Basic Eng..

[38] Matsumoto J., Morrow D., Kaufman K., Davis D., Ahlskog J.E., Walker A., Sneve D., Noseworthy J., Rodriguez M. (2001). Surgical therapy for tremor in multiple sclerosis: An evaluation of outcome measures. Neurology.

[39] Patel V.V. (2020). Ziegler-Nichols Tuning Method. Resonance.

